# Assessment of Tanning Beds in 3 Popular Gym Chains

**DOI:** 10.1001/jamanetworkopen.2019.18058

**Published:** 2019-12-20

**Authors:** Sherry L. Pagoto, David E. Conroy, Kelsey Arroyo, Jared Goetz, Ashley B. West, Samantha Mulcahy, Molly E. Waring

**Affiliations:** 1 Department of Allied Health, University of Connecticut, Storrs; 2 Department of Kinesiology, Pennsylvania State University, University Park; 3 School of Health Sciences, Oakland University, Rochester, Michigan

## Abstract

This cross-sectional study examines the proportion of gyms from 3 large national chains in the United States that offer tanning services, the number of tanning beds offered by these gyms, and regional differences in the number of gyms that provide tanning services.

## Introduction

The proliferation of gym chains across the United States increases US individuals’ access to physical activity. Unfortunately, some gym amenities used to attract consumers are harmful, namely, tanning beds. Tanning bed use is reported to be a risk factor for malignant melanoma.^1^ A systematic review reported an association between indoor tanning and physical activity.^2^ Indoor tanning among physically active people is concerning, given the reported association between physical activity and melanoma risk.^3^

Little is known about how pervasive the presence of tanning beds is in gyms. The present cross-sectional study examined the proportion of gyms from 3 popular US gym chains that have tanning beds, the number of tanning beds offered, and regional differences in the number of gyms with tanning beds.

## Methods

All locations from 3 of the 6 largest US national gym chains (Anytime Fitness, Gold’s Gym, and Planet Fitness) in 33 states and Washington, DC, were sampled across all 5 geographic regions of the US (Northeast, Southeast, Midwest, Southwest, and West). We extracted data on locations from websites and searched each gym name with a state abbreviation on Google Maps. Three of us (K.A., J.G., and A.B.W.) and 2 raters (A. Argueta and M. Campbell) independently verified all gyms. Between June 2018 and February 2019, each gym was contacted by telephone up to 3 times to ask whether they offered tanning and, if so, how many tanning beds were available for use. We included 7 of 11 states in the West, 3 of 4 states in the Southwest, 8 of 12 states in the Midwest, 7 of 11 states in the Northeast, and 9 of 12 states in the Southeast (including Washington, DC). We compared availability of tanning beds by chain and region using χ^2^ tests followed by pairwise comparisons. We used analysis of variance followed by pairwise comparisons comparing total and mean number of tanning beds per gym by chain and region. The institutional review board of the University of Connecticut determined that this study does not constitute human participant research, and therefore approval and informed consent were waived. This study followed the Strengthening the Reporting of Observational Studies in Epidemiology (STROBE) reporting guideline for cross-sectional studies. Data were collected between June 2018 and February 2019 and were analyzed in May 2019.

## Results

Three gym chains had 1927 locations in 33 states and Washington, DC. Anytime Fitness had the most locations (n = 1067), followed by Planet Fitness (n = 732) and Gold’s Gym (n = 128). Contact rates were 98% for Gold’s Gym (126 of 128), 99% for Planet Fitness (727 of 732), and 82% for Anytime Fitness (874 of 1067) for a total of 1727 gyms reached. Calls to Anytime Fitness were often directed to a call service and more frequently went unanswered.

Overall, 78% of the gyms (1347 of 1727) reached had tanning beds, which exceeded the rate reported in Canada (43% [88 of 203]).^4^ A greater proportion of Planet Fitness gyms (99% [726 of 727]) had tanning beds than Anytime Fitness (65% [569 of 874]; *P* < .001) and Gold’s Gym (41% [52 of 126]; *P* < .001) (Table). A total of 4660 tanning beds were found in 1347 gyms. Planet Fitness provided more tanning beds than the other gyms (Planet Fitness, 3736 tanning beds; mean [SD] tanning beds per gym, 5.14 [1.9]; 95% CI, 5.0-5.20 vs Anytime Fitness, 798 tanning beds; mean [SD] tanning beds per gym, 0.97 [0.86]; 95% CI, 0.91-1.03; *P* < .001) (Table), accounting for 80% (3736 of 4660) of total tanning beds. The Midwest had the highest proportion of gyms with tanning beds (87% [446 of 514]; 95% CI, 84%-90%; *P* < .001) (Table).

**Table.** Prevalence and Number of Tanning Beds in Gyms by Chain and Region

Facility and Region	No.	Presence of Tanning Beds, No. (%) [95% CI]	*P* Value	Tanning Beds, Total No.	No. of Tanning Beds per Gym, Mean (SD) [95% CI]	*P* Value
Total gyms reached	1727	1347 (78) [76%-80%]	NA	4660	2.79 (2.54) [2.72-2.86]	NA
Gym chain						
Anytime Fitness	874	569 (65) [62%-68%]	<.001	798	0.97 (0.86) [0.91-1.03]	<.001
Planet Fitness	727	726 (99) [99%-100%]		3736	5.14 (1.96) [5.08-5.20]	
Gold’s Gym	126	52 (41) [33%-50%]		126	1.07 (2.54) [0.63-1.51]	
Region						
Northeast^a^	353	259 (73) [69%-78%]	<.001	1011	2.93 (2.38) [2.78-3.08]	.02
Southeast^b^	506	381 (75) [72%-79%]		1476	3.00 (2.72) [2.86-3.14]	
Midwest^c^	514	446 (87) [84%-90%]		1390	2.73 (2.72) [2.59-2.87]	
Southwest^d^	164	116 (71) [64%-78%]		372	2.34 (2.13) [2.12-2.56]	
West^e^	190	145 (76) [70%-82%]		411	2.51 (1.95) [2.32-2.70]	

Abbreviation: NA, not applicable.

^a^ Maine, New Hampshire, Vermont, Massachusetts, Connecticut, Rhode Island, and Pennsylvania.

^b^ North Carolina, Delaware, Alabama, Georgia, Mississippi, West Virginia, Maryland, Kentucky, and Washington, DC.

^c^ Michigan, Wisconsin, North Dakota, South Dakota, Nebraska, Missouri, Iowa, and Kansas.

^d^ Arizona, New Mexico, and Oklahoma.

^e^ Alaska, Hawaii, Colorado, Idaho, Montana, Washington, and Wyoming.

## Discussion

A total of 4660 tanning beds were found across 33 states and Washington, DC, with the highest concentration being in the Midwest. Gyms that offer tanning services may be perpetuating the notion that tanning is part of a healthy lifestyle, which undermines public health warnings about the dangers of indoor tanning.

Limitations of this study include the focus on only 3 gym chains. These gyms are among the 6 largest US gym chains, among which only 1 other has tanning beds (SNAP Fitness). The other 2, LA Fitness and 24 Hour Fitness, do not have tanning beds at any locations according to their corporate offices. Only 33 states and Washington, DC, were studied, which represents 41% of gyms from these chains nationwide. Some gyms could not be reached despite numerous attempts, but our response rate of 90% was high.

Gyms can circumvent the federal 10% tanning tax by not charging per visit^5^; thus, future tanning legislation should address tanning beds in diversified business environments. Gym chains make exercise accessible and affordable to the public; however, clinicians should counsel patients who use gyms to avoid tanning beds.

## References

[1] Vogel RI, Ahmed RL, Nelson HH, Berwick M, Weinstock MA, Lazovich D Exposure to indoor tanning without burning and melanoma risk by sunburn history. J Natl Cancer Inst. 2014;106(6):dju112. doi:10.1093/jnci/dju219 24872541 PMC4081627

[2] Heckman CJ, Manning M The relationship between indoor tanning and body mass index, physical activity, or dietary practices: a systematic review. J Behav Med. 2019;42(2):-. doi:10.1007/s10865-018-9991-y 30446921 PMC9092438

[3] Moore SC, Lee IM, Weiderpass E, Association of leisure-time physical activity with risk of 26 types of cancer in 1.44 million adults. JAMA Intern Med. 2016;176(6):816-825. doi:10.1001/jamainternmed.2016.1548 27183032 PMC5812009

[4] Huang CM, Kirchhof MG A cross-sectional study of indoor tanning in fitness centres. J Cutan Med Surg. 2017;21(5):401-407. doi:10.1177/1203475417706059 28418712

[5] Internal Revenue Service Excise tax on indoor tanning services frequently asked questions. https://www.irs.gov/businesses/small-businesses-self-employed/excise-tax-on-indoor-tanning-services-frequently-asked-questions. Accessed July 8, 2019.

